# Modelling the effects of CO_2_ on C_3_ and C_4_ grass competition during the mid-Pleistocene transition in South Africa

**DOI:** 10.1038/s41598-020-72614-2

**Published:** 2020-10-01

**Authors:** Michaela Ecker, Douglas Kelley, Hiromitsu Sato

**Affiliations:** 1 Institute of Pre- and Protohistoric Archaeology, University of Kiel, Kiel, Germany; 2 Archaeology Centre, University of Toronto, Toronto, Canada; 3 UK Centre for Ecology and Hydrology, Wallingford, UK; 4 Department of Earth Sciences, University of Toronto, Toronto, Canada

**Keywords:** Palaeoecology, Environmental sciences

## Abstract

Palaeoenvironmental reconstructions of the interior of South Africa show a wetter environment than today and a non-analogous vegetation structure in the Early Pleistocene. This includes the presence of grasses following both C_3_ and C_4_ photosynthetic pathways, whereas C_3_ grasses decline after the mid-Pleistocene transition (MPT, c. 1.2–0.8 Ma). However, the local terrestrial proxy record cannot distinguish between the potential drivers of these vegetation changes. In this study we show that low glacial CO_2_ levels, similar to those at the MPT, lead to the local decline of C_3_ grasses under conditions of decreased water availability, using a vegetation model (LPX) driven by Atmosphere–Ocean coupled General Climate Model climate reconstructions. We modelled vegetation for glacial climates under different levels of CO_2_ and fire regimes and find evidence that a combination of low CO_2_ and changed seasonality is driving the changes in grass cover, whereas fire has little influence on the ratio of C_3_:C_4_ grasses. Our results suggest the prevalence of a less vegetated landscape with limited, seasonal water availability, which could potentially explain the much sparser mid-Pleistocene archaeological record in the southern Kalahari.

## Introduction

**Figure 1:**
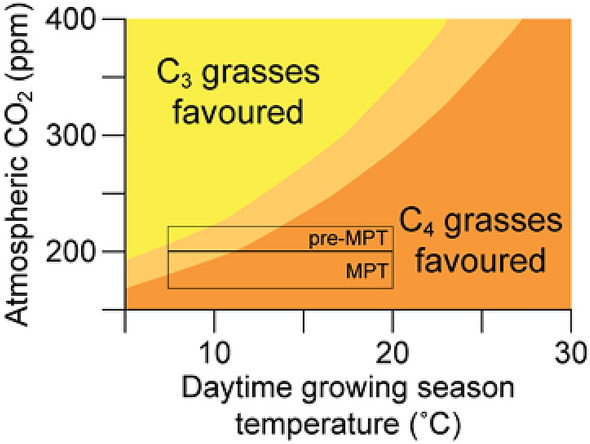
Prediction of atmospheric CO_2_ and daytime growing season temperature conditions that favour C_3_ or C_4_ grasses. Black box covers CO_2_ levels and potential temperatures during glacials in the central interior of South Africa before the Mid-Pleistocene transition (‘40 k world’) and during the Mid-Pleistocene transition (‘100 k world’) (adapted after^11^ and^12^).

South Africa can be divided into three seasonal rainfall zones (winter, summer and year-round rainfall), which in turn strongly influence the distribution of vegetation^1^ (Supplementary Fig. S3). Palaeoenvironmental studies hypothesized that areas that are in the summer rainfall zone of the savanna biome might have been under the influence of an expanding winter rainfall zone during Pleistocene glacial conditions^2–4^. This could have led to shifts in the vegetation structure of the savanna biome, which today consists broadly of tropical and subtropical grasses following the C_4_ photosynthetic pathway with scattered small trees and bushes following the C_3_ photosynthetic pathway^1^. Rainfall seasonality, in turn, is a major control of fire^5^, and regular wildfire is common in African savannas, enabling turnover in the grass layer. C_4_ grasses, on the whole, tend to recover faster after fires than C_3_ grasses. This may be due to rapid regrowth due to generally higher photosynthetic rate, especially with the removal of shading from higher canopies post-fire^6^. The importance of fire in maintaining modern ecosystems has been shown in modelling studies^7^. Fire intensity and regularity is challenging to reconstruct for the Pleistocene, particularly when microcharcoal records are absent. However, there can be considerable overlap between C_3_ and C_4_ species of grass^8^. Atmospheric CO_2_ levels are another major control of plant photosynthesis. C_3_ plants, which can also include temperate grasses, thrive under high CO_2_ levels, whereas C_4_ grasses are more efficient under low CO_2_ levels (Fig. 1). In laboratory studies, for example, glacial CO_2_ has been found to increase the stomatal conductance of C_4_ grasses to greater effect than C_3_ grasses, while maintaining its high water-use efficiency^9^. This difference in water-use efficiency is a likely mechanism for C_4_ grasses to outcompete C_3_ grasses in arid regions. The vegetation response to changing CO_2_ is therefore heavily dependent on local subsurface conditions^10^.

Approximately 900,000 years ago, ice ages switched from occurring every 41 kyr to every 100 kyr, called the mid-Pleistocene transition (MPT), which in turn had consequences for the global CO_2_ record. Boron isotope reconstructions of the global CO_2_ records during the MPT (c. 1.2–0.8 Ma) show glacial (c. 160–200 ppm) CO_2_ levels to be particularly low; lower than in the preceding Early Pleistocene (c. 185–250 ppm). At the same time interglacial CO_2_ levels are similar before and after the MPT (c. 250–320 ppm), but on average lower during the MPT (c. 200–300 ppm)^13^. CO_2_ reconstructions from the oldest ice in Antarctica confirm lower glacial CO_2_ concentrations during the MPT in comparison to the 40 kyr world before and the 100 kyr world afterwards^14^. In total, the glacial-interglacial span of CO_2_ increased substantially. In accordance with their physiology, going from, on average, glacial 185–250 ppm to 160–200 ppm has a much larger effect on photosynthesis in C_4_ plants than on C_3_ plants (Fig. 1).

Recent studies suggested early human evolution during the Pliocene and Pleistocene in non-analogous, productive C_3_ plant dominated environments in parts of Africa^15,16^. In the central interior of South Africa, Wonderwerk Cave is the site with the longest and most fine-grained record of palaeoenvironmental change, covering the last two million years^17^. This site and other local multi-proxy records showed an environment during the Early Pleistocene (c. 1.96–0.78 Ma) that included the presence of persistent standing bodies of water and grasses following both C_3_ and C_4_ photosynthetic pathways^18,19,20^ (Supplementary Table S1). After c. 800 kyr, C_3_ grasses show a decline in abundance at Wonderwerk Cave and are not a part of the local vegetation during the Holocene, as is common in African savannas. Grazer enamel isotopes and short cell grass phytoliths records show changing proportions of C_3_ and C_4_ grasses but cannot distinguish between the drivers and their interactions.

Dynamic Global Vegetation Models (DGVMs), which simulate climate-driven vegetation processes, can generate spatially-continuous reconstructions of past environments to complement discrete and dispersed proxy records. They also allow insight into past biosphere–atmosphere interactions using the body of ecophysiological theory embedded in DGVMs. However, it is critical that model output be regarded in the context of independent palaeoecological records to build confidence in their findings. After knowing the state of past environments, it is imperative that we identify and firmly understand the processes that led to their formation. In this study, we use the LPX-DGVM^21^ driven by Atmosphere–Ocean coupled General Climate Model (AOGCM) climate output^22^, to test three possible drivers forcing vegetation change that have been suggested in the Wonderwerk Cave palaeoenvironmental study^18^: (1) atmospheric CO_2_ levels, (2) rainfall seasonality, and (3) disturbance through fire. Under identical glacial climate reconstructions from four different AOGCM outputs, we modulated CO_2_ levels to determine its effect on vegetation cover, particularly the expansion or contraction of C_3_ and C_4_ grasses. We test if the proposed drivers were influencing the changes that have been reconstructed from proxy records for the Early- and mid-Pleistocene in the southern Kalahari at c. 27° southern latitude. Model runs were performed under 150 ppm (post-MPT scenario) and 250 ppm (pre-MPT scenario) glacial CO_2_ conditions, with and without the possibility of natural fires occurring (Supplementary Fig. S1 and S2). We used extreme values on both ends for the CO_2_ values to explore the maximum sensitivity of the system. From the results, we estimate the effect of CO_2_ concentration on C_3_/C_4_ grass cover, determine the climatic niches of C_3_/C_4_ grasses and elucidate ecophysiological mechanisms to explain low CO_2_-mediated C_4_ grass expansion.

**Figure 2:**
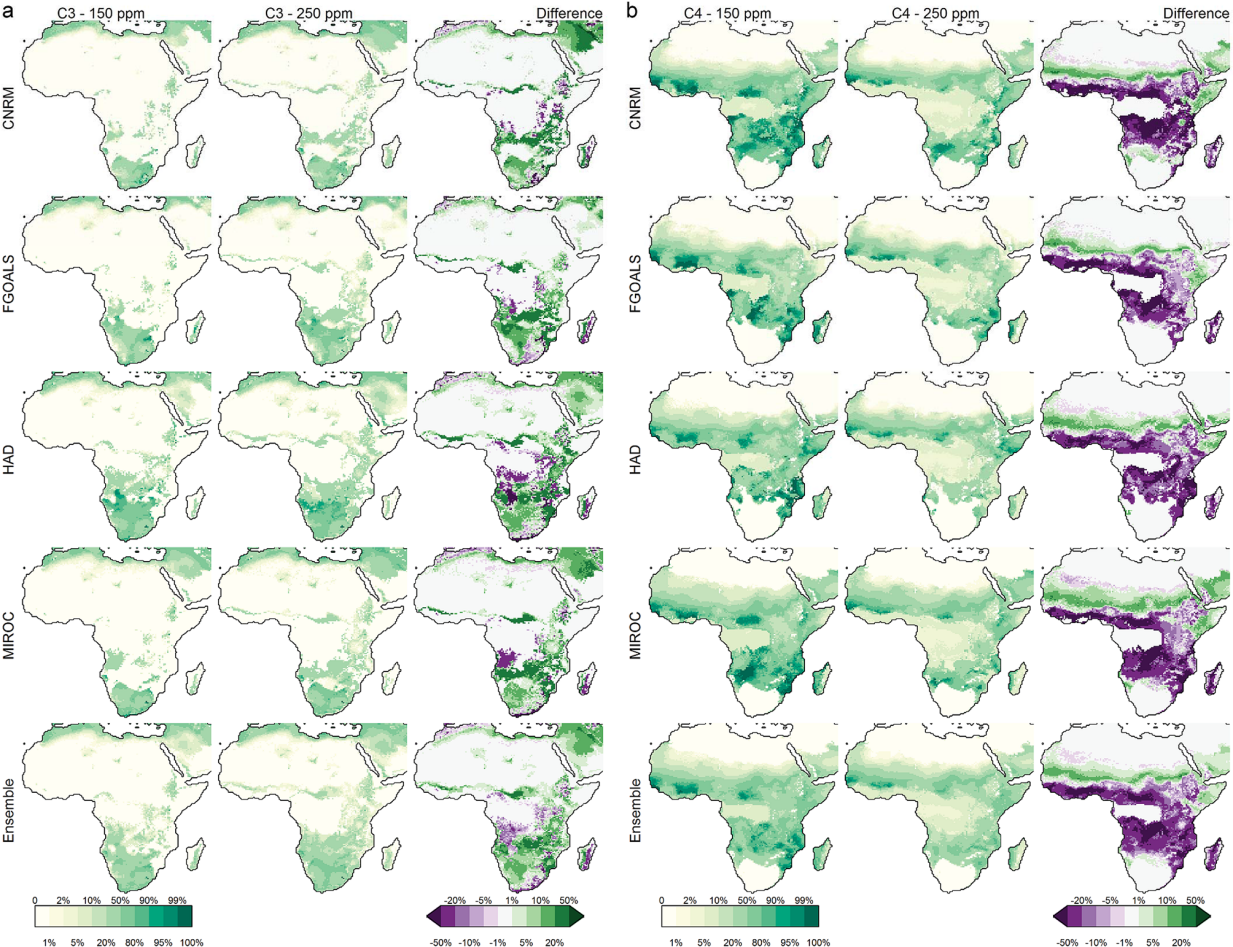
Foliage projective cover of (**a**) C_3_ and (**b**) C_4_ grasses at 150 ppm and 250 ppm, and their difference derived from vegetation reconstructions simulated by LPX-DGVM^21^. In the difference plot, green indicates increases and purple indicates decreases. All models are shown with the impact of fire on in the model. The figure was constructed using raster2.8–19 (https://CRAN.R-project.org/package=raster) and mapproj1.2.6 (https://CRAN.R-project.org/package=mapproj) in R 3.5.2 (https://www.R-project.org/). The present-day coastline was obtained from mapsv3.3.0 (https://CRAN.R-project.org/package=maps), while LGM coastlines are based on data provided by PMIP2^22^.

**Figure 3:**
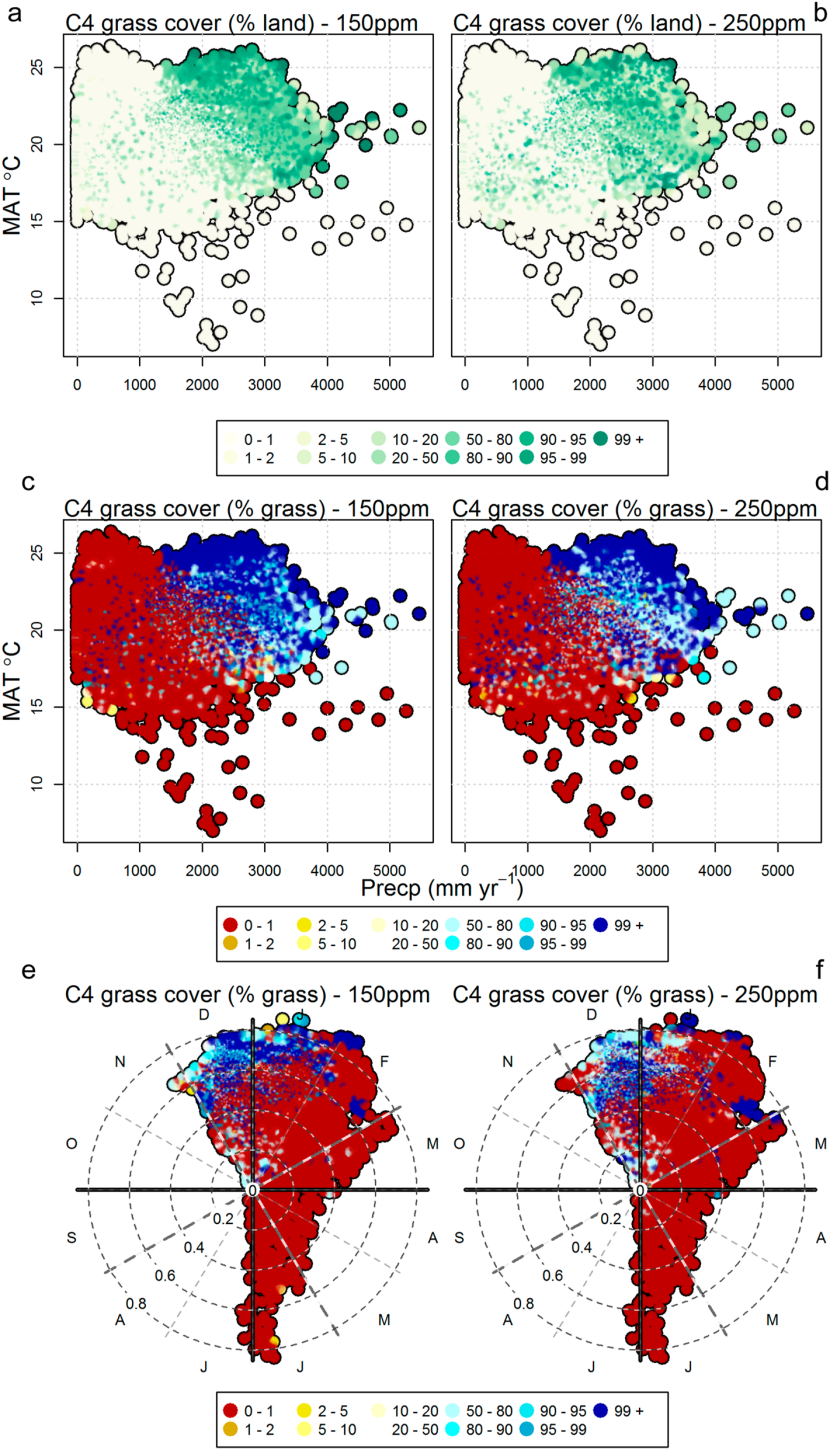
C_4_ grass cover, with fire on, in climate space as simulated by LPX-DGVM^21^. Left is 150 ppm runs, right 250 ppm runs. (**a**–**d**) MAP (x-axis) versus MAT (y-axis), (**e**–**f**) seasonal concentration (distance from centre) and phase (direction) based on^23^. Higher concentration means shorter rain season. Colours are: (**a**,**b**) C_4_ grass coverage as % of land (**c**–**f**) % of grass that is C_4_.

Consistent with our hypothesis, model reconstructions suggest that CO_2_ levels have a clear influence on C_4_ grass cover over Africa. However, C_4_ grass cover is most significantly impacted over East Africa and to a smaller degree over Southern Africa (Fig. 2). For all four LGM climate reconstructions, the low CO_2_ (150 ppm) scenario, tends to expand and increase the density of C_4_ grass cover. This trend is consistent for runs with and without fire, suggesting that fire has little influence on the competition between grass types within the model in this context (Supplementary Fig. S1 and S2). The large difference in C_4_ grass cover particularly around the central African regions is a result of reduced tree cover instead of direct competition with C_3_ grass, as tree cover is sensitive to fire regime changes. There is significant inter-climate model variation in Southern Africa, where the FGOALS-g1.0 reconstructions show clearest an increase in the western half of Southern Africa in C_3_ grass cover and decrease in the eastern half of Southern Africa in the pre-MPT scenario compared to the post-MPT scenario (Fig. 2). In contrast, the other models show a more uniform increase in C_3_ grass cover due to low CO_2,_ except for the eastern coastal areas (Fig. 2). These differences can be traced back to details in rainfall and temperature differences in the models, as shown in Supplementary Fig. S3. Figure 3 shows grass cover in the climate space of mean annual temperature (MAT) and mean annual precipitation (MAP), showing that C_4_ cover tends to increase when MAT is above 15 °C and MAP is above 500 mm/year in areas dominated by concentrated summer rainfall. At low CO_2_, this border becomes more prominent, while at the same time, less aridity is needed for a shift to occur.

The modelling results match well with the palaeoenvironmental changes seen in interior South Africa^18^. In the early Pleistocene, dry and wet phases with both C_3_ and C_4_ grasses exist. After the MPT, evidence for increased seasonality focussed on summer rainfall^18^ and significant groundwater changes in the Kuruman hills area^19,20^ indicate increasing aridity. In these arid, low glacial CO_2_ conditions C_3_ grasses, as with all C_3_ plants, were more stressed than C_4_ plants. On long timescales, C_4_ grasses may have outcompeted C_3_ grasses, which do not return in significant numbers during the Pleistocene^24,25^. Low levels of CO_2_ during glacials alone may not have led to major changes in vegetation in the interior of South Africa during the Pleistocene. However, the combined effects of low CO_2_ in addition to changes in the growing season could have pushed vegetation through certain bioclimatic thresholds (Fig. 3). C_4_ plants require on average over c. 22 °C growing season (warmest month) temperature to effectively compete against C_3_ plants, limiting their distribution to warm tropical and subtropical areas^11,26,27^. Below this ‘crossover temperature’, C_3_ photosynthesis has a higher quantum yield^11^. In glacial periods during the Pleistocene, when temperatures were lower in all seasons, the growing season in South Africa might have shifted when winter rainfall influences extended east^2–4^. This would increase water availability at Wonderwerk Cave year-round, which could then support a mix of C_3_ and C_4_ grasses, as exists today in a very small area in the southern Karoo, close to the year-round rainfall zone^28^. In addition, lower temperatures mean less evapotranspirative demands on plants, resulting in lower water stress. Changes in rainfall seasonality or amount alone without CO_2_ changes cannot explain the long-term change to C_4_ grass savannas as there is no return of a substantial amount of C_3_ grasses in the humid late Pleistocene at Wonderwerk Cave^24^. Our results have implications for hominin responses during the later phase of the Early Stone Age as seen in the rich Acheulean cultural record in the southern Kalahari, which contrasts with a much sparser mid- and late Pleistocene record^24,29^. The much sparser archaeological record could be a reaction to the less vegetated landscape (less C_3_ plants) and limited, seasonal water availability. Major changes in the cultural record of the region, for example, the transition from the Early to the Middle Stone Age (c. 500–200 kyr), occur not at the start but during and after this ongoing environmental change.

**Figure 4:**
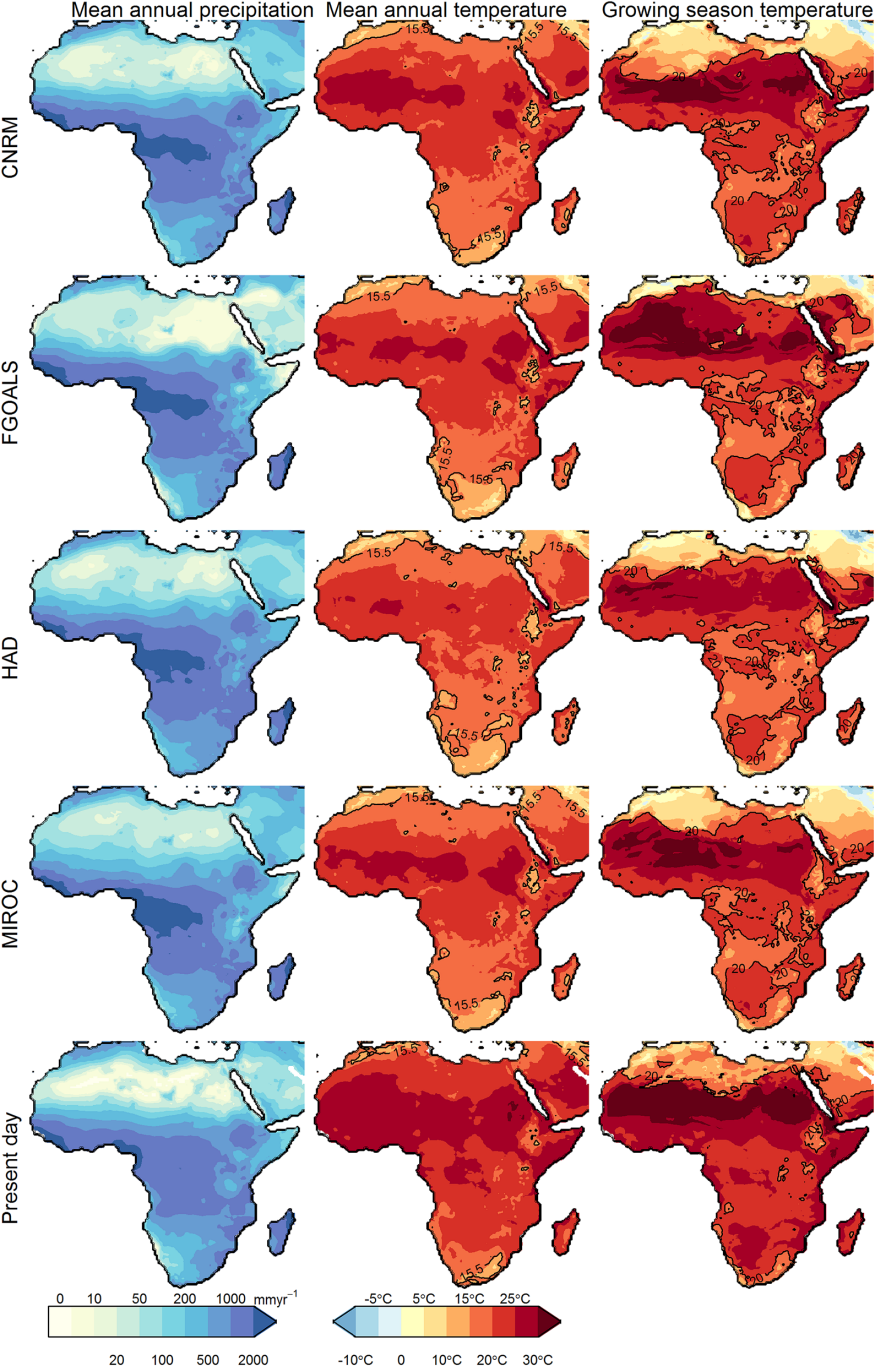
Mean annual precipitation, mean annual temperature and temperature during peak rainfall. Peak rainfall is equivalent to the growing season. Present day climate conditions taken from CRU TS 4.01^34^. Peak rainfall is based on the month of the phase (Supplementary Fig. S3). Contours at mean annual temperatures of 15.5 °C and peak rainfall temperatures of 20 °C. The figure was constructed using raster 2.8–19 (https://CRAN.R-project.org/package=raster) and mapproj1.2.6 (https://CRAN.R-project.org/package=mapproj) in R 3.5.2 (https://www.R-project.org/). The present-day coastline was obtained from mapsv3.3.0 (https://CRAN.R-project.org/package=maps), while LGM coastlines are based on data provided by PMIP2^22^.

Our study is an example of testing local terrestrial proxy reconstructions, as other areas of Africa clearly follow different trajectories (Fig. 2). Eastern parts of South Africa^30,31^ and East Africa^32,33^, have a dominant C_4_ grass component before the MPT. The impact of low CO_2_ on the already existing C_4_ grass cover here is shown as substantial in our modelling results. Our study shows how vegetation models are able to test hypotheses generated from local palaeoecological research targeting specific drivers and their interactions. However, any model output is always a reflection of larger patterns rather than a site-specific environmental reconstruction. It is noteworthy that in all reconstructions, C_4_ grasses tend not to appear south of ~ 18°S, where C_3_ grasses dominate regardless of CO_2_ level (Fig. 2), even though C_4_ grasses dominate the landscape there today^26^. One reason is possibly the different winter rainfall expansion between the models, and C_3_ grasses being a result of winter rainfall influence. However, the models only simulate winter rainfall for the far south-west of the continent (Fig. 4, Supplementary Fig. S3). Another could be the models lower temperature limits for C_4_ photosynthesis (see “Methods”) interacting with possible local temparure biases in climate model simulation. More attention has to be paid in future work on such discrepancies between vegetation models and modern environmental data. In turn, this can be used to refine the models for use in past as well as in future climate change predictions.

## Methods

*Model* description The Land surface Processes and eXchanges (LPX) is a coupled, process-based fire-enabled dynamic global vegetation model (DGVM). Full details of the dynamic vegetation component can be found in Sitch et al. (2003)^35^, while the fire model is described in Thonicke et al. (2010)^36^ and Prentice et al.^21^. LPX simulates atmosphere-to-biosphere interactions and ecosystem structure and function; computing spatially and temporally resolved estimates of potential vegetation cover and height, biomass, soil carbon and water and energy fluxes. LPX uses nine Plant Functional Types (PFT) to represent potential vegetation. PFTs compete within grid cells, where their differential ecophysiological responses to driving climate data, background turnover (or mortality) and resistance to fire disturbance determine their relative abundances. PFTs can be either tree or grasses. Trees are split by climate range (tropical, temperate, boreal), leaf type (broadleaf, evergreen), and phenological response (evergreen, raingreen, summergreen) and grasses are split between by C_3_ and C_4_ photosynthetic pathways.

To represent CO_2_ fertilization, LPX explicitly couples CO_2_ assimilation with transpiration using the photosynthesis-water balance scheme^37,38^ adapted from the Farquhar model^39,40^. Available CO_2_ reduces potential water stress on a plant by lowering the required stomatal conductance (g_c_) for a given photosynthetic rate. Maximum potential stomatal conductance (g_cMax_) for when water is not limiting depends on the maximum potential day-time pathway-specific photosynthetic assimilation rate (A_max_) and minimum canopy conductance parameters, and atmospheric CO_2_ concentration. If gcMax requires a transpiration rate (D) that is greater than the available water supply (S, which is a function of soil water content and soil properties), then g_c_ (and therefore photosynthesis and production) is reduced in such a way as to be consistent with an empirical formulation derived from Monteith (1995)^41^. At higher atmospheric CO_2_ concentrations, g_cMax_ decreases while A_max_ remains the same, and the value of S that induces water stress is, therefore, lower and maximum production rates can occur at lower moisture availability. Furthermore, when S is less than D, g_c_ (and therefore production) requires less down-regulation. Increased CO_2_ thus leads to a fertilization effect, with increases production in drier conditions.

The distribution of C_3_ versus C_4_ plants is dynamically determined by light-use competition between the two photosynthetic pathways^35^. The ratio of internal to ambient CO_2_ concentrations optimal for photosynthesis is lower in C_4_ compared to C_3_, thereby reducing photorespiration and making photosynthesis more efficient. It also means that C_4_ productivity is less sensitive to the lowering of atmospheric CO_2_ concentrations. Leaf respiration as a fraction of Rubisco capacity is, however, higher for C_4_ photosynthesis.

Under plentiful water supply and present-day CO_2_, C_4_ plants tend to have higher Gross Primary Production (GPP) than C_3_ plants at temperatures above ~ 20 °C^36^. Increased water stress, induced by dry conditions or reduced CO_2_ concentrations, tends to reduce this crossover temperature. Under cooler temperatures, the simulated higher metabolic costs of C_4_ photosynthesis outway advantages in photorespiration, and C_3_ gains a competitive edge, Additionally, C_3_ has no lower temperature limit for survival—through reduced productivity will effectively prevent establishment at very low temperatures, this is well those considered in this study. C_4_ grasses, however, do not survive temperatures of less than 15.5 °C.

Fires occur from lightning ignitions, and the probability of an ignition event causing a fire depends on local fuel and atmospheric moisture content. Fire spread, flame height and residence time are based on weather conditions and fuel moisture, calculated using the Rothermel equations^42^. The area affected by fires is the product of the number of fires and the average spread of fire. LPX simulates fire mortality through two pathways: leaf and crown scorching, affecting all PFTs and cambial damage, affecting just tree PFTs. The amount of crown scorching depends on the height and intensity of the fire in relation to the height of the local vegetation. The probability of mortality from crown scorch increases as flame height increases beyond the canopy height of each PFT. While the fire mortality in this scheme will kill the shorter grass PFTS more than tree PFTs, grass PFTs tend to recover faster. Depending on other environmental conditions, water supply and CO_2_ concentration, differing growth rates will preferentially select either C_3_ and C_4_ grass PFTs.

*Modelling Protocol* We follow the same modelling protocol as^43^. Four distinct reconstructions of LGM climate (MIROC3.2, HadCM3M2, FGOALS-g1.0, CNRM-CM33)^44^, generated by four atmosphere–ocean general circulation models (AOGCM) derived from the paleoclimate model intercomparison project 2 (PMIP2), were used to drive the Land surface Processes and eXchanges (LPX) DGVM^21^. Vegetation was ‘spun-up’ from bare ground for 4000 years to reach equilibrium from which model runs were set for an additional 1380 years. Raw model output was then post-processed to show distributions of vegetation and climate-vegetation relationships as per^43^.

*Seasonal comparison* We use season phase and concentration metrics from^23^. Each month, m, was represented by a vector with direction (m) corresponding to the time of year and the length corresponding to the magnitude of the variable for that month. A mean vector was calculated the average of x and y vectors:

$$\begin{aligned} & \theta_{m} = 2\left( {m - 1} \right)/12 \\ & x = {\Sigma }_{{\text{m}}} \left[ {v_{m} \times {\cos}\left( {\theta_{m} } \right){ }} \right] \\ & y = {\Sigma }_{{\text{m}}} \left[ {v_{m} \times {\text{sine}}\left( {\theta_{m} } \right){ }} \right] \\ \end{aligned}$$

The ratio of the mean vector length to annual average described the seasonal concentration (C) of the variable, timing is described by the mean direction (P):

1 $$C = \sqrt {x^{2} + y^{2} /{\Sigma }_{{\text{m}}} v_{m} }$$

2 $$P = arctan\left( {x/y} \right)$$

C is equal to 1 when the variable is 0 in all but 1 month (i.e., there is only 1 month of rainfall when assessing rainfall seasonality) and P is equal to that month. C is 0 and P is undefined when the variable is evenly distributed throughout the year.

## Supplementary information

Supplementary Information.

## Data Availability

Available from the authors on request.

## Code availability

Available from the authors on request.
