# Supplementary material for: Modelling the effects of CO2 on C3 and C4 grass competition during the mid-Pleistocene transition in South Africa
Source: Sci Rep. 2020 Oct 1;10:16234. doi: 10.1038/s41598-020-72614-2 (PMC7530989; doi:10.1038/s41598-020-72614-2)
Supplement: Supplementary file 1 — Supplementary Information. [file 41598_2020_72614_MOESM1_ESM.pdf]

Supplementary Online Materials for

Modelling the effects of CO_2 on C_3 and C_4 grass competition during the mid-Pleistocene transition in South Africa

Michaela Ecker*, Douglas Kelley, Hiromitsu Sato

*correspondence to: mecker@ufg.uni-kiel.de

This file includes:

Supplementary Figures 1-3
Supplementary Table 1
References

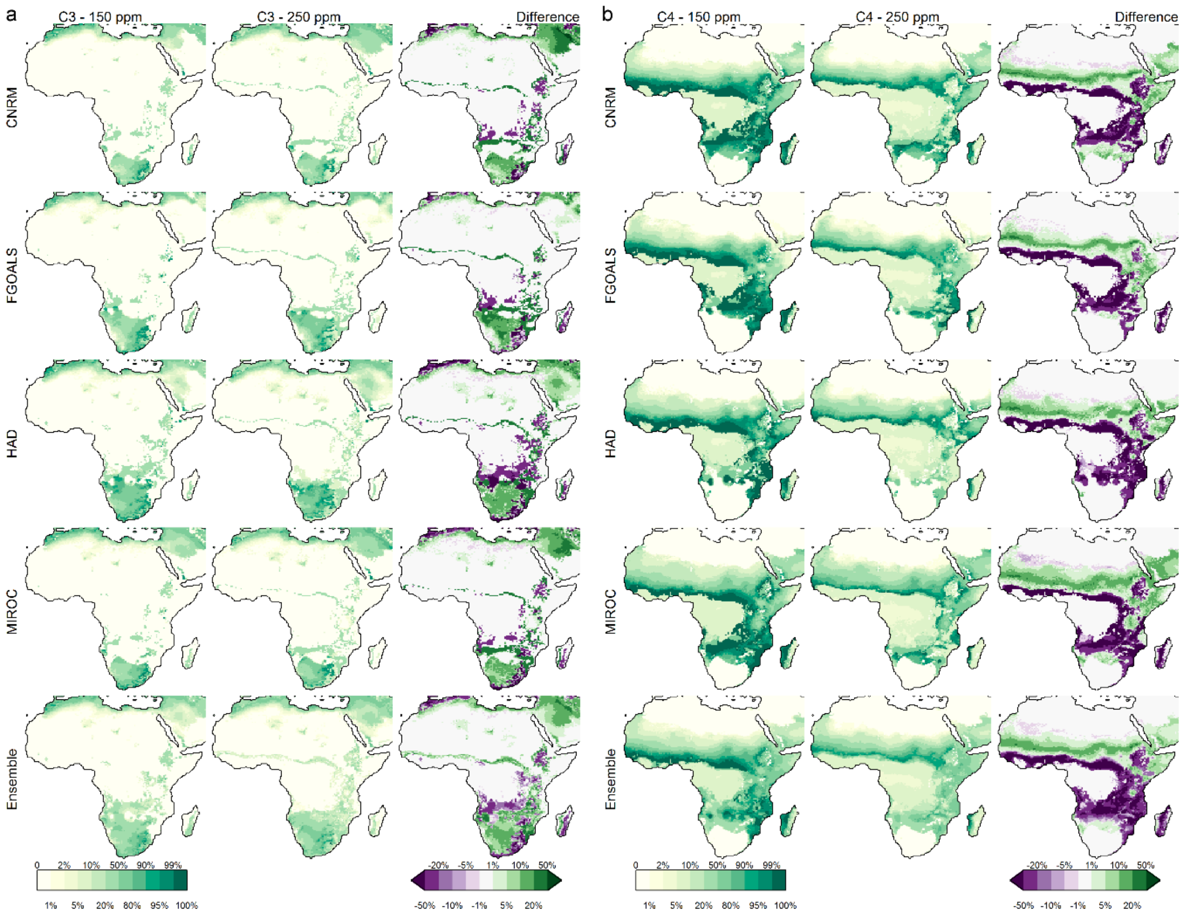

Figure S1: Foliage projective cover of (a) C_3 and (b) C_4 grasses at 150 ppm and 250 ppm, and their difference derived from vegetation reconstructions. In the difference plot, green indicates increases and purple indicates decreases. All models are shown without any impact of fire. The figure was constructed using raster2.8-19 (https://CRAN.R-project.org/package=raster) and mapproj1.2.6 (https://CRAN.R-project.org/package=mapproj) in R 3.5.2 (https://www.R-project.org/). The present-day coastline was obtained from mapsv3.3.0 (https://CRAN.R-project.org/package=maps), while LGM coastlines are based on data provided by PMIP2[22].

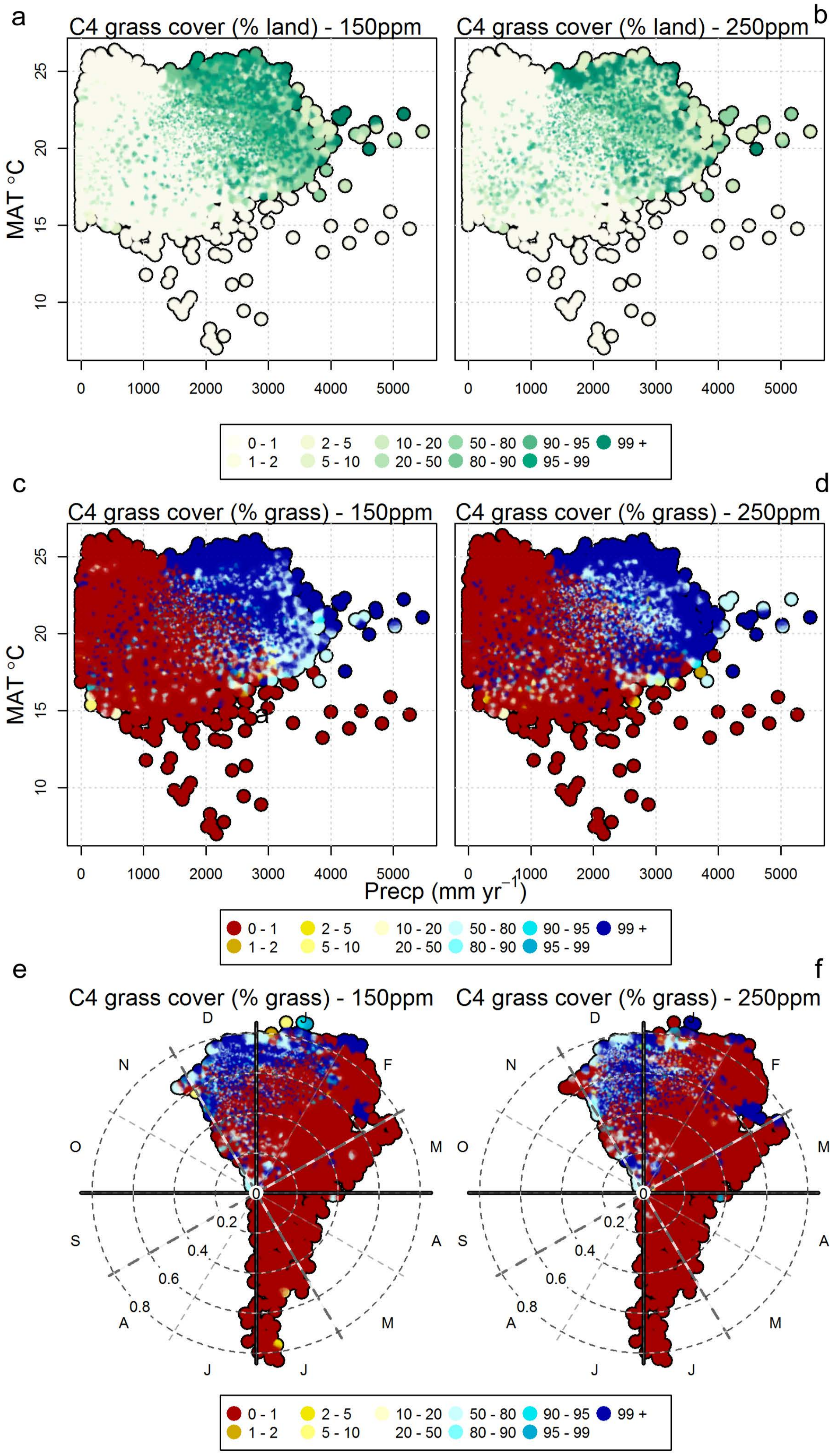

Figure S2: C_4 grass cover, with fire off, in climate space as simulated by LPX-DGVM[21]. Left is 150ppm runs, right 250 ppm runs. a-d) MAP (x-axis) vs MAT (y-axis), e-f) seasonal concentration (distance from centre) and phase (direction) based on[23]. Higher concentration means shorter rain season. Colours are: a-b) C_4 grass coverage as % of land c-f) % of grass that is C_4.

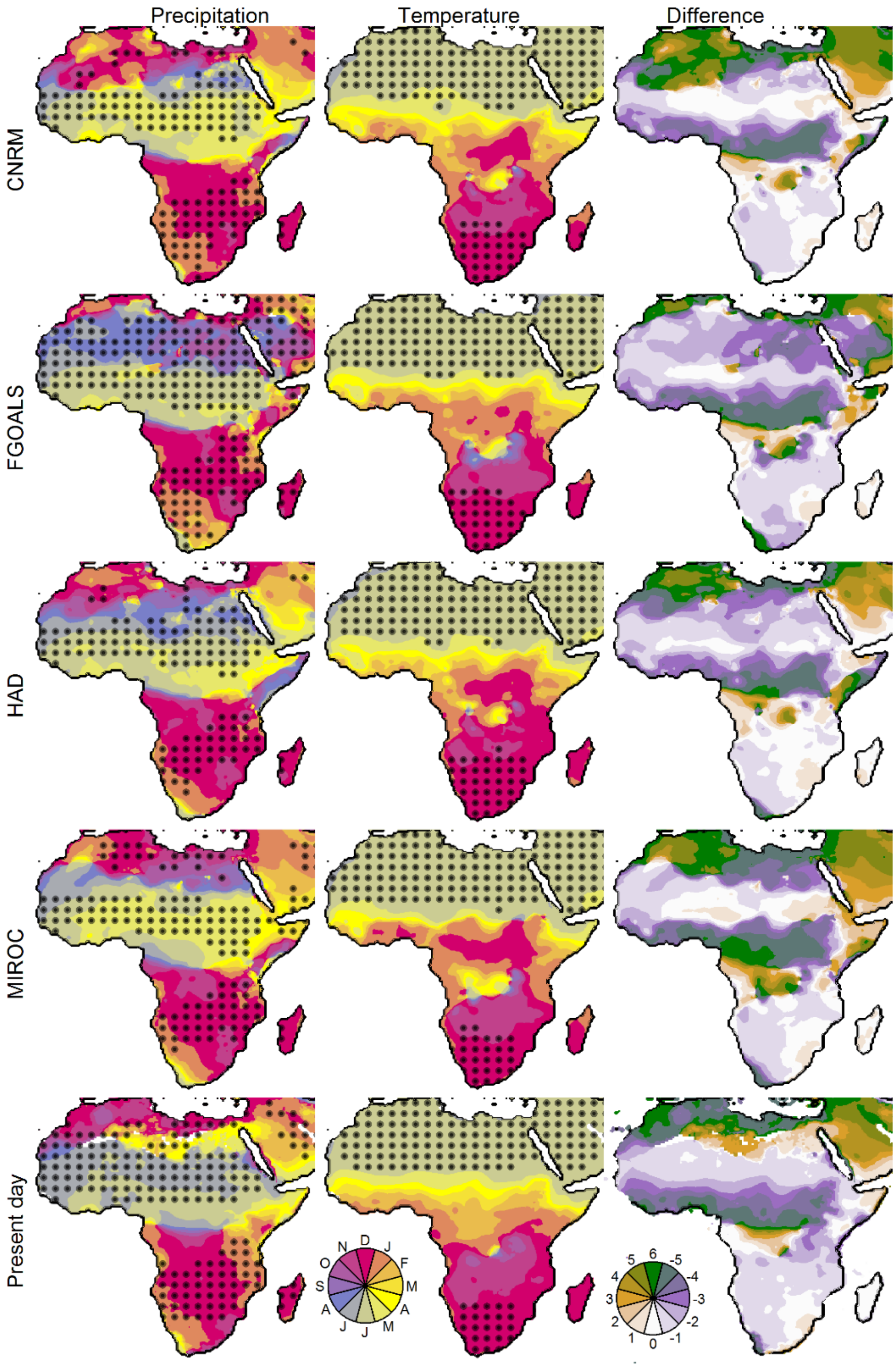

Figure S3: Phase and concentration of rainfall season and temperature. Phase of season shown in colour, concentrated seasons are shown with dots. Areas without dots in rainfall are associated with year-round rainfall. The difference plot shows discrepancies in phase between rainfall and temperature in months – white is where peak rainfall and temperature occur at the same time - i.e where summer rainfall dominates. Green is where rainfall and temperature are out of phase, i.e where winter rainfall dominates. Brown is areas of spring rainfall (i.e temperature lags rain), and blue is autumn rainfall (rainfall lags temperature). This figure was created with the same software as Figure 3, with the addition of plotrix3.7-4 (https://CRAN.R-project.org/package=plotrix).

Table S1: Wonderwerk Cave stratigraphy, dating, associated lithics and palaeoproxy reconstruction for the Early Stone Age strata from excavation area 1. OES = ostrich eggshell. Based on [18, 45-50].

**Strata & Age**	**Lithic technology**	**Environment**	**Climate**	**Proxy evidence**
**Strata 6-8** (older than 350kyr)	Late Acheulean	**Spread of C_4 grassland, loss of C3 grasses**	**Semi-arid, summer rainfall, dry spells**	OES $\delta^{18}O$, enamel $\delta^{18}O$ and $\delta^{13}C$, microfauna, macrofauna, eggshell biometry
**Stratum 9** 0.99-0.78 Ma	Late Acheulean	**C_3 and C_4 grasses**	**Semi-arid, summer rainfall**	enamel $\delta^{18}O$ and $\delta^{13}C$, OES $\delta^{18}O$, phytoliths
**Stratum 10** 0.99-1.07 Ma	Acheulean	**C_3 and C_4 grasses, less open; first extreme C_4 values in diet**	**Fluctuating arid/humid phases, overall more humid**	enamel $\delta^{18}O$ and $\delta^{13}C$, OES $\delta^{18}O$, micromorphology, phytoliths, eggshell biometry, marcofauna, mircofauna
**Stratum 11** 1.07-1.78 Ma	Acheulean	**Shrubland/ Woodland with C_3 and C_4 grasses**	**Cold, humid, winter rainfall influence**	enamel $\delta^{18}O$ and $\delta^{13}C$, OES $\delta^{18}O$ and $\delta^{13}C$, phytoliths, microfauna, macrofauna

**Stratum 12**<br>1.78-1.96 Ma	Oldowan	**Mixed C_3-C_4 savanna, open**	**Semi-arid, summer rainfall, warm**	OES $\delta^{18}O$ and $\delta^{13}C$, enamel $\delta^{18}O$ and $\delta^{13}C$, phytoliths, microfauna, macrofauna

References:

45. Brink, J., Holt, S. & Horwitz, L.K. The Oldowan and early Acheulean Mammalian Fauna of Wonderwerk Cave (Northern Cape Province, South Africa). *Afr. Archaeol. Rev.* ***33***(3), 223-250 (2016).

46. Chazan, M. *et al.* The Oldowan horizon in Wonderwerk Cave (South Africa): archaeological, geological, paleontological and paleoclimatic evidence. *J. Hum. Evol.* ***63***, 859–866 (2012).

47. Ecker, M., Botha-Brink, J., Lee-Thorp, J.A., Piuz, A. & Horwitz, L.K. Ostrich eggshell as a source of palaeoenvironmental information in the arid interior of South Africa: A case study from Wonderwerk Cave in *Changing Climates, Ecosystems and Environments within Arid Southern Africa and Adjoining Regions (ed.* Runge, J.), *Palaeoecol. Afr.* **33**, 95-115 (2015).

48. Fernandez-Jalvo, Y., & Avery, D. M. Pleistocene micromammals and their predators at Wonderwerk Cave, South Africa. *Afr. Archaeol. Rev.* ***32***(4), 751-791 (2015).

49. Goldberg, P., Berna, F. & Chazan, M. Deposition and Diagenesis in the Earlier Stone Age of Wonderwerk Cave, Excavation 1, South Africa. *Afr. Archaeol. Rev.* **32**, 613-643 (2015).

50. Rossouw, L. An early Pleistocene phytolith record from Wonderwerk cave, northern Cape, South Africa. *Afr. Archaeol. Rev*. ***33***(3), 251-263 (2016).
